# Effectiveness of Social Behaviors for Autonomous Wheelchair Robot to Support Elderly People in Japan

**DOI:** 10.1371/journal.pone.0128031

**Published:** 2015-05-20

**Authors:** Masahiro Shiomi, Takamasa Iio, Koji Kamei, Chandraprakash Sharma, Norihiro Hagita

**Affiliations:** Intelligent Robotics and Communication Laboratories, ATR, Kyoto, Japan; Politehnica University of Bucharest, ROMANIA

## Abstract

We developed a wheelchair robot to support the movement of elderly people and specifically implemented two functions to enhance their intention to use it: speaking behavior to convey place/location related information and speed adjustment based on individual preferences. Our study examines how the evaluations of our wheelchair robot differ when compared with human caregivers and a conventional autonomous wheelchair without the two proposed functions in a moving support context. 28 senior citizens participated in the experiment to evaluate three different conditions. Our measurements consisted of questionnaire items and the coding of free-style interview results. Our experimental results revealed that elderly people evaluated our wheelchair robot higher than the wheelchair without the two functions and the human caregivers for some items.

## Introduction

### Attitudes of elderly people toward robotic daily care

Due to the steep increase in the number of elderly people in countries as Japan, Italy, and Germany, assisting them has become one of the essential purposes of robotics. Recent attitude surveys in Japan reported that more than 60% of elderly people positively evaluated the robots those which provided physical assistance [1, 2]. These results suggest Japanese people would be more acceptive of robotics technologies for elderly support than other countries such as USA and EU [3, 4]. Actually there are several support robots which are developed in Japan, as reported in the survey papers [5, 6]. One main reason for such positive evaluations is that elderly people feel less hesitation in requesting physical support from a robot than a human caregiver, echoing past research which also concluded that humans feel less hesitation in making high workload task requests to a robot than a person [7]. This suggests the validity of the attitude survey’s results [1, 2]. Less hesitation increases the perceived ease of use, which is one essential factor for *intention to use* in elderly people [8, 9] for support robots.

Robotics researchers have focused on physical support for elderly people including several works on walking aids with robotics technologies [10, 11]. Many researchers have used wheelchair robots for moving support; various wheelchair robots and simulators were well surveyed by Faria et al. [12]. Kobayashi et al. focused on safe autonomous and simultaneous navigation for multiple wheelchair robots [13]. Other research works focused on the user interfaces for wheelchair robots, such as voice interface [14] and brain machine interface [15]. Morales et al. proposed a comfortable navigation method by considering human perceptions [16]. Freire et al. have also developed a modality-independent interface for an autonomous wheelchair robot for people with distinct levels of disability [17]. How et al. have investigated the performance of a semi-autonomous wheelchair robot for elderly people with cognitive impairments [18].

Moreover, in this context, several researchers have considered the World Health Organization’s International Classification of Functioning, Disability and Health (ICF). The ICF is used to explain the state of function and health of people. Therefore, use of the ICF makes it easy to understand physical capabilities of elderly people, and is helpful to find the needs of each person with different disability. For example, Tanaka et al. have developed and evaluated assistive robots which are designed for people with disabilities, by considering the ICF [19]. Vidal et al. also considered the ICF in order to develop the sound-based environment recognition system which helps in distinguishing different environments for people with disabilities [20].

However, these research works missed to compare robotic and human support, they did not evaluate feelings of elderly as perceived intention to use. Piau et al. showed the importance of exploring the needs of elderly people, in particular frail people, through the reviews of gerontechnologies for aging society [21]. From this perspective, actual use of robots by elderly people to investigate their feeling would be important, but in the attitude surveys in Japan no experiments involving actual robots were performed prior to the surveys [1, 2]. Therefore, it remains unclear whether elderly people really have intention to use a current robot system for daily care. Their attitude might change if their assumptions toward robot systems are too different from the real settings. If elderly people have unrealistic expectations of a robot’s capabilities, their attitude toward daily care robots might become negative.

Based on this context, it is important to investigate the feelings of elderly people who have actually used a real robot system to establish a basis for the use of robots for elderly care. For this purpose, we focused on a moving support robot, i.e., an autonomous wheelchair robot, mainly for two reasons. Firstly, based on the ICF Model, health condition of a person is related to his body function, activity and participation; and where moving support is strongly related to all of these factors. Understanding of such an essential support with robotics technologies would be important for addressing use of technology in elderly care. Secondly, It is one common area where elderly people worldwide require support or physical assistance and various robots have already been developed for moving support. Investigating feelings about moving support with robotics technologies would have impact on those research and development works. Inline with these reasons, we posed the following research question:

RQ1: Do elderly who experienced a robotic moving support perceive higher intention to use for a wheelchair robot than human caregivers?

### Social behaviors for moving support robots for seniors

Social behavior of robots those support elderly is a key factor of their intention to use. Our past research works showed that in a shopping assistance task, which is one kind of physical support for elderly people, such social behaviors as simple chatting, which is not always necessary for such tasks, increase the intention to use in users [22]. This research work also reported that social behaviors increase the perceived ease of use and enjoyment, which are related factors to intention to use. We expect that social behaviors, which should be designed based on the observations of caregivers while pushing wheelchairs for moving support, are important to increase the intention to use for autonomous wheelchair robots.

However, past research related to moving support has mainly focused on developing robust and safe navigation for wheelchair robots without focusing on such social behaviors [12, 13, 16]: in other words, theme has been to design a wheelchair robot as a tool for elderly people. But it is appropriate to consider intention to use for elderly support robots. For example, some people reported that a robot’s human-likeness might increase hesitation to use for some specific tasks [7, 22]. These research works suggest that social behaviors, which are related to the human-likeness of the robots, have a risk which negatively affects the intention to use. These considerations suggest the following open question:

RQ2: Should an autonomous wheelchair robot behave socially in ways that are considered easy or relaxing by elderly people in moving support?

### Hypotheses and predictions

This study answers two research questions.

Concerning RQ1, we expect that elderly will feel higher intention to use for autonomous wheelchair robots than human caregivers in moving support, as reported in attitude surveys [1]. Because they feel less hesitation for such physical support from robots than human caregivers, this increases the perceived ease of use, which is a related factor to intention to use. Based on these considerations, we made the following hypothesis:

**Prediction 1** Elderly people will feel higher intention to use toward the wheelchair robot than human caregivers.

Concerning RQ2, we have two conflicting expectations. The first is the positive effects of the social behaviors of a wheelchair robot. As past research work showed [22], the social behaviors of robots increase its perceived ease of use and enjoyment, which are related to intention to use.

The second one is the negative effects of the social behaviors of a wheelchair robot; previous research works about moving support for elderly people designed their robots as a tool without social behaviors like caregivers [10, 11]. This approach might be appropriate to fulfill the “intention to use” of a wheelchair robot from a tool perspective; i.e., a tool-like design is better for wheelchair robots that support elderly people. Based on these considerations, we established two contradictory predictions:

**Prediction 2-a** Elderly people will feel higher intention to use for a wheelchair robot with social behaviors than one without them.

**Prediction 2-b** Elderly people will feel lower intention to use for a wheelchair robot with social behaviors than one without them.

To address these two research questions, we observed the social behaviors of caregivers while pushing a wheelchair at a private resident home and implemented them on an autonomous wheelchair robot.

## Social behavior design based on observation of caregivers

To design social behaviors for an autonomous wheelchair robot, we visited a private resident home where about 100 seniors live, among whom about 20 rely on wheelchairs every day. We observed the caregiver’s behaviors while they pushed wheelchairs for a day and interviewed the facility’s administrator. We identified two kinds of social behaviors, individual-oriented and place-oriented, which can be implemented on an autonomous wheelchair robot to an extent.

### Individual-oriented behaviors

#### Greeting by name

Most caregivers greet the elderly people by name when they start pushing the wheelchair. Such interaction is essential to construct relationships between caregivers and elderly people. It is also important for human-robot interaction; in fact, in our previous studies, we found that people appreciated robots that called their names [23, 24]. Moreover, other research have also investigated the effects of calling people by name regarding acceptance of robots [25]. Therefore, we continued this strategy and prepared behaviors to greet the seniors by names: “Hello, Yamada-san.”, this behavior is used before starting the moving support.

#### Preferred speed

Most caregivers adjusted their wheeling speed to match the individual preferences of the seniors. In particular, experienced caregivers moved more slowly during moving supports. However, maintaining slow speed consistently is generally difficult for humans; for example, some caregivers increase their speed when they are busy. In an interview, the administrator explained the gap between the wheeling speed preferred by the seniors and the preferred speed by the caregivers. The administrator also pointed out that caregivers try to move slowly during wheeling, but many seniors wanted them to move even slower. Recently, Morales et al. have investigated the importance of preferred velocity for wheelchair users from viewpoint of their comfort [26]. Therefore, we defined each elderly person’s preferred speed so that the robot can always easily adhere to slow speeds or adapt to individual preferences.

### Place-oriented behaviors

In our observations, we found that caregivers often commented about the location or arrival at or wait for elevators, entry to the cafeteria, the recreation room, or their own rooms. For example, after arriving in front of the elevator, a caregiver might say, “Now, let’s get onto the elevator..” Even though the seniors rarely responded to this announcement, the caregivers continued to talk to them. Such speaking behaviors relay caregiver’s intentions to the elderly and might also decrease their anxiety. We prepared behaviors to provide information about such specific places as “this spot is relatively narrow.” These behaviors are used before going to pre-defined places that have different properties.

## Methods

### Ethics statement

This research was approved by the Advanced Telecommunications Research Institute’s ethics committee for studies involving human participants. Written, informed consent was obtained from all the participants in our study.

### Participants

In the experiment, 28 elderly people participated: 14 women and 14 men, whose age averaged 74.0 years, S.D 6.85. 10 people who required daily care are living in private residential care homes. Five routinely use wheelchairs, and other five sometimes use wheelchair when they need to move long distance. Two senior staff members from the care homes also participated in the experiment to support the seniors who are living in the home. The remaining 18 participants did not require daily care; among those only one participant had experiences with wheelchair during a hospitalization incident.

### Environment

**Fig 1:**
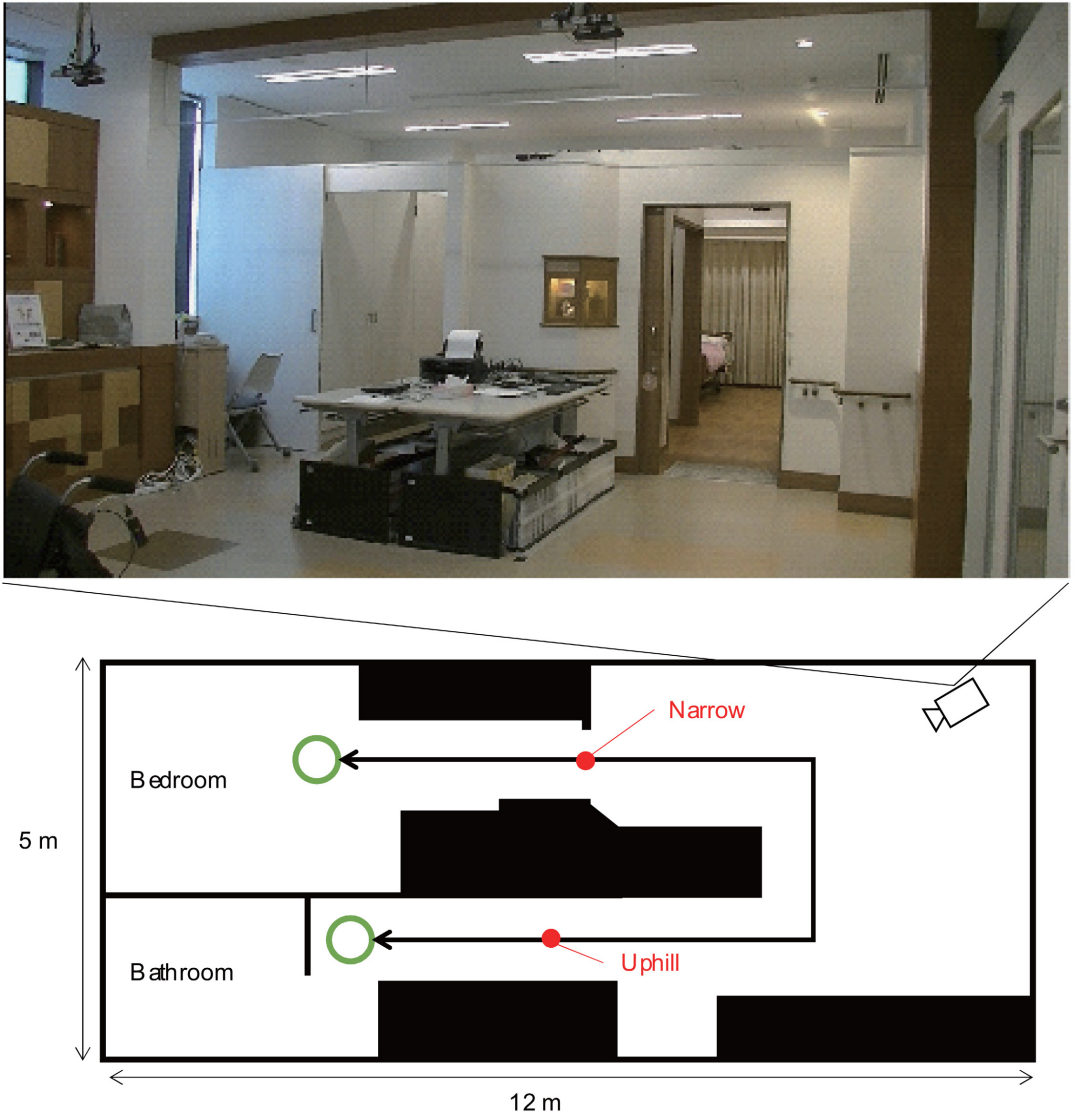
Experimental settings.

We conducted our experiment in an experimental residential care home facility that consisted of a bedroom and a bathroom, because a round trip between the bedroom and the bathroom is typical situation of using wheelchair at care homes. The room sizes and designs are identical to the actual care home where some of the participants are living. Fig 1 shows a map of the environment and the route between the bedroom and the bathroom.

### Procedure

The participants were given a brief description of our wheelchair robot. We also provided information on its nature and its safety functions. Each participant rode in the wheelchair robot and determined her preferred speed by choosing from three robot speeds: 300, 600, or 900 mm/sec. 600 mm/sec was the average speed at which the caregivers pushed the elderly people, as measured in the private resident home. Seven participants preferred 300 mm/sec, 19 preferred 600 mm/sec, and two chose 900 mm/sec. After investigating the preferred speed of each individual, we conducted the experiment and prepared the following three conditions to answer our research questions:

- Simple condition: after selecting a target location, the wheelchair robot automatically moved with the participant on it at 600 mm/sec and did not say anything; no social behaviors were performed.
- Social condition: the wheelchair robot greeted elderly person using her/his name, and also automatically moved after selecting a target location and talked based on the robot’s location. We adjusted the speed to match each participant’s preferred speed which was measured at the beginning of the experiment. We set two kinds of place information in this environment: a narrow place at the bedroom’s door and a slope in front of the bathroom. When the robot is approaching these places, it repeats pre-defined text: “this place is relatively narrow” before passing the bedroom’s door, and “now we are going slightly uphill” at the beginning of the slope.
- Caregiver condition: the caregiver wheeled the participants in the usual manner (Fig 1), this condition reproduces the daily situation of seniors who use wheelchairs and need help in moving around. For the participants living in the care home, a staff member wheeled them during the experiment in the usual fashion; we did not specify moving speed or conversation behavior. For the rest of the participants, the experimenter wheeled them at around 600 mm/sec and talked to them using similar contents as the social condition, around a narrow place at the bedroom’s door and a slope in front of the bathroom.

The experiment had a within-participant design. Each participant participated in three sessions of different conditions. The order of the conditions was counterbalanced. Staff members remained in the environment for safety and to record videos. The participants filled out a questionnaire after each session. Since participants were difficult to get on and off frequently from the wheelchair robot due to their physical limitations, they filled out questionnaire on the wheelchair at the same environment.

### Robot

**Fig 2:**
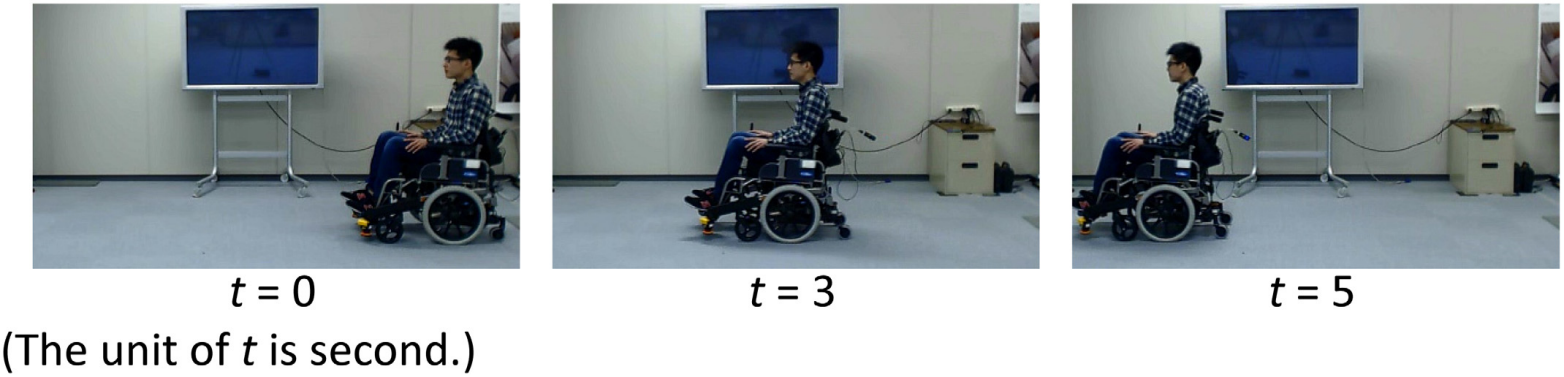
Wheelchair robot. The individuals in this manuscript gave their written informed consent (as outlined in the PLOS consent form) to publish these case details.

We used a 59-cm wide, 85-cm tall, and 104-cm long differential drive robotic wheelchair from Nissin Medical Industries (NEO-PR45, Fig 2). Note that this wheelchair is a commercial electric wheelchair, therefore ordinary people can buy and use it for their daily life. The hardware specs are similar to other commercial electronic wheelchairs. Therefore, its properties e.g., appearance and design were not special for this experiment. For safety reasons, we set it to move at a maximum linear velocity of 900 *mm*/*sec* and 30 *degree*/*sec* rotational velocity, and the accelerations for the forward and rotational movements was 600 *mm*/*sec* ^2^ and 30 *degree*/*sec* ^2^, respectively. We installed three laser range finders (Hokuyo UTM-30LX) on the wheelchair for localization and obstacle detection.

We applied a time-varying dynamic window (TVDW) [27] for navigation. To localize the robot position, we employed a particle-filter based localization mechanism [28] and speech synthesis software called XIMERA [29] for the robot’s speech. The system generally operated autonomously. If the localization function failed, a human operator corrected the error in the localization. More details of system information are described in [30].

For speed adaption, the wheelchair robot changes its moving speed using the preferred speed information, which was measured in advance. For the speaking behavior function, the robot makes pre-defined small talk based on its position and registered map information around narrow spaces and slopes. It also uses the names of the seniors in the wheelchair and announces departures and arrivals.

## Measurements

We measured the intention to use to investigate whether elderly people wanted to use the wheelchair robot more than the human caregivers and whether they preferred the robot who engaged in social behaviors. The measurement of “intention to use” consisted of three items [8], but we only used one because the participants and the staff members from the care homes requested to shorten the questionnaires due to their limited availability at the experiment site.

We measured the degree of ease to make a request and the comfort during locomotion because past research work reported that such ease of use is a source of the intention to use [8]. Since two factors represent the perceived ease of use in moving support, we also measured the degree of perceived enjoyment, which is also a source of intention to use [8]. Finally, we measured the total evaluation of the moving support to investigate the complete attitude of the elderly people for moving support. Following questionnaire items were evaluated on a 1-to-7 Likert scale.

- Intention to use (ITU)
- Degree of easiness to make a request (ER)
- Degree of comfort
- Degree of enjoyment
- Total evaluation

We also interviewed the participants to gather their opinions about each condition. We asked them to explain their ratings for each item after completing the questionnaires.

## Results

### Verification of hypotheses

**Fig 3:**
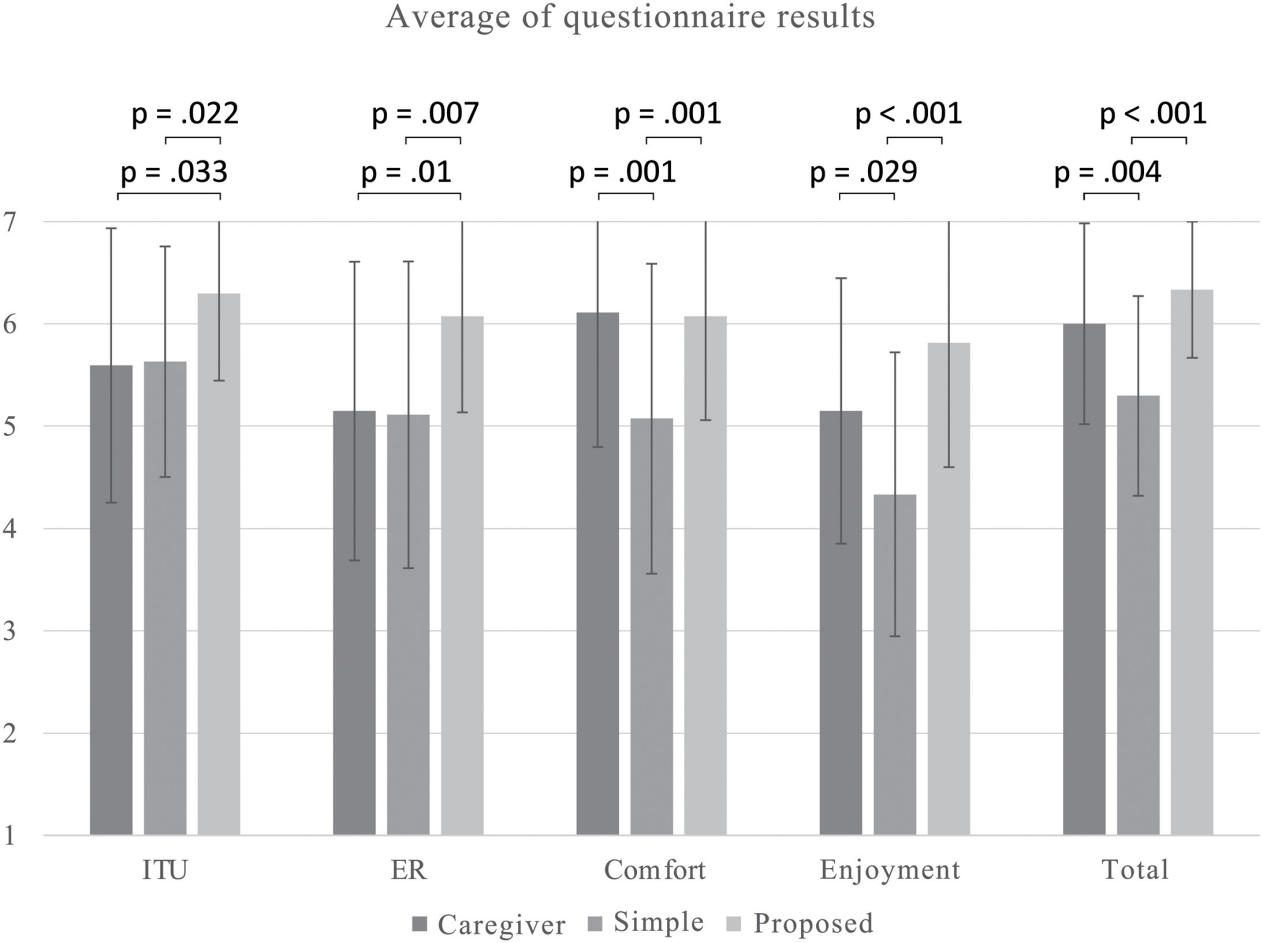
Questionnaire results.

Fig 3 shows the result of each questionnaire item. Since one male participant could not participate in all three conditions, we eliminated his questionnaire items from the evaluations (S1 Table includes the questionnaire data). We conducted a one-factor within subject ANOVA for each item. Table 1 compares the statistical results.

**Table 1:** Questionnaire results.

	Simple		Social		Caregiver				
	M	SE	M	SE	M	SE	f	p	*partial* *η* ^2^
ITU	5.630	1.149	6.296	0.869	5.593	1.366	4.908	.011	.159
ER	5.111	1.528	6.074	0.958	5.148	1.486	6.538	.003	.201
Comfort	5.074	1.543	6.074	1.035	6.111	1.340	10.419	<.001	.286
Enjoyment	4.333	1.414	5.815	1.242	5.148	1.432	12.026	<.001	.316
Total	5.296	0.993	6.333	0.679	6.000	1.000	13.326	<.001	.339

For the “intention to use”, we found a significant difference among the conditions (*F*(2,52) = 4.908, *p* = .011, *partial* *η* ^2^ = .159). Multiple comparisons with the Bonferroni method revealed significant differences: social > simple (*p* = .033) and social > caregiver (*p* = .022). No significance was found between caregiver and simple (*p* = 1.0).

Contrary to prediction 1, the questionnaire results from the experiments indicate that the wheelchair robot without social behaviors is not significant from the caregivers in terms of “intention to use.” Prediction 1 was not supported.

Regarding prediction 2, the questionnaire results indicate that the wheelchair robot with social behaviors was evaluated more significantly than the caregivers in terms of “intention to use.” Prediction 2-a was supported.

### Related scales of intention to use and total evaluation

To investigate more details about perception of the participants about moving support, we also analyzed the degree of easiness to make a request and the degrees of comfort and enjoyment and conducted a one-factor within subject ANOVA for each item. Table 1 compares the statistical results.

For the “degree of easiness to make a request,” we found a significant difference among the conditions (*F*(2,52) = 6.538, *p* = .003, *partial* *η* ^2^ = .201). Multiple comparisons with the Bonferroni method revealed significant differences: social > simple (*p* = .007) and social > caregiver (*p* = .01). No significance was found between caregiver and simple (*p* = 1.0).

For the “degree of comfort,” we found a significant difference among conditions (*F*(2,52) = 10.419, *p* <.001, *partial* *η* ^2^ = .286). Multiple comparisons with the Bonferroni method revealed significant differences: caregiver > simple (*p* = .001) and social > simple (*p* = .001). No significance was found between caregiver and social (*p* = 1.0).

For the “degree of enjoyment,” we found a significant difference among the conditions (*F*(2,52) = 12.026, *p* <.001, *partial* *η* ^2^ = .316). Multiple comparisons with the Bonferroni method revealed significant differences: caregiver > simple (*p* = .029) and social > simple (*p* <.001). A significant trend was found between social and caregiver (*p* = .096).

For the “total evaluation,” we found a significant difference among the conditions (*F*(2,52) = 13.326, *p* <.001, *partial* *η* ^2^ = .339). Multiple comparisons with the Bonferroni method revealed significant differences: caregiver > simple (*p* = .004) and social > simple (*p* <.001). No significance was found between caregiver and social (*p* = 0.33).

Regarding prediction 1, the questionnaire results of the wheelchair robot without social behaviors were significantly lower than the caregivers in “the degree of comfort,” “the degree of enjoyment,” and “the total evaluations’, opposite to our hypothesis. The rest of items did not show significant differences between conditions. Thus, these results did not support prediction 1.

Regarding prediction 2, the questionnaire results indicate that the wheelchair robot with social behaviors showed a significant difference in terms of “the degree of easiness to make a request.” Other measurements are not significant and generally resemble those of the caregivers. We thought that these differences would increase the perceived ease of use, and the “intention to use” would also become higher than the caregiver condition. Thus, these results support prediction 2-a.

### Interviews

We analyzed the interview results to investigate the reasons behind the different impressions among the conditions. Two coders analyzed and classified the transcribed interview results. We gathered 118 sentences of their impressions (S2 Table includes the coded data). Cohen’s kappa coefficient [31] from the classifications of the two coders was 0.726 and yielded the following:

1. I could more easily request moving support from the robot than the humans.
2. I could more easily request moving support from the humans than the robot.
3. The robot was safe for moving support.
4. The robot was not safe for moving support.
5. I liked/wanted speaking behaviors from the robot.
6. I did not like/want speaking behaviors from the robot.
7. The robot’s locomotion capability was adequate.
8. The robot’s locomotion capability was inadequate.
9. The locomotion capability of the humans was adequate.
10. The locomotion capability of the humans was inadequate.
11. Others

Table 2 shows the number of each category in each condition. These results support our predictions. For example, the interview results from the caregiver and social conditions showed that elderly people explained the easiness to request moving support to the robot with social behaviors. Interestingly, only a few seniors reported an easiness to make a request in the simple condition.

**Table 2:** Summarization of interview results.

	Caregiver	Simple	Social
I could more easily request moving support from the robot than the humans.	12	2	11
I could more easily request moving support from the humans than the robot.	0	2	1
The robot was safe for moving support.	1	2	0
The robot was not safe for moving support.	2	4	5
I liked/wanted speaking behaviors from the robot.	2	10	19
I did not like/want speaking behaviors from the robot.	1	2	7
The robot’s locomotion capability was adequate.	4	0	1
The robot’s locomotion capability was inadequate.	4	7	7
The locomotion capability of the humans was adequate.	6	0	0
The locomotion capability of the humans was inadequate.	2	0	0
Others	2	0	2

Elderly people seemed to feel more anxiety or discomfort about the robot locomotion capabilities than the caregivers. The autonomous wheelchair did not collide with anything during the experiments, but the accelerations or the turning locomotions are different from those done by the humans; this might explain their opinions. In fact, the statistical results of the related questionnaire item (i.e., the degree of comfort) were not significant.

Even though most of our participants positively evaluated the robot’s speaking behaviors, only one senior explicitly evaluated the call by name behavior in the interview. Moreover, only one senior explicitly hesitated about the speaking behaviors: “It was annoying because I already understood the surrounding situations without the robot speech,” a few other seniors complained about the robot voice.

Even if some negative interview results are shown, these results support prediction 2-a. Several negative interview results under the simple condition might show why prediction 1 was not supported.

## Discussion

### Implications

This study shows a promising way for use of an autonomous wheelchair robot for moving support of elderly people. Implemented social behaviors are relatively simple but are designed based on observations of human caregivers. These behaviors actually were important to increase social acceptance by elderly people. These results would suggest that more social behaviors of robots are important for future use. For example, in this study we limited interactions between elderly people and the robot due to its limited sensing capabilities; but if the robot has a robust speech recognition system or a person identification system, the robot could interact more naturally with not only a user but also surrounding people such as other elderly people or staff. Involving other people to interaction between the robot and the user would be important to increase opportunities for social participation of the users..

Moreover, if multiple autonomous wheelchair robots are used in simultaneously at the same environment, such interactions with other people become more important. For example, when a wheelchair robot will be crossing with others, their avoidance behaviors and greeting behaviors would be important. If its behaviors are not human-like, i.e., different from assumptions of other people, they might feel stress to live with robots in the same environments. Such behaviors should be considered when two wheelchair robots will be crossing too. In other words, behaviors to increase social acceptance by surrounding people are also needed.

### Ethical consideration

Using robots for supporting elderly people is imbued with ethical considerations in various fields of application. For example, such works are counter to elderly exepectations to receive services from humans, and force them to receive services from machines. In caregivers’ opinions, moving support from an autonomous wheelchair robot might decrease opportunities for rehabilitation; if seniors became dependent on such a robot and stop moving by themselves, their own physical activity will decrease.

On the other hand, several machines have already assumed the main role in elderly care to support both caregivers and elderly people. For example, patient-lifts transfer people from one spot (e.g., bed) to another (e.g., chair). We believe that physical support situations should be aided by such systems because the physical loads are difficult; actually supporting caregivers is another important issue in super-aging societies. Moreover, using autonomous wheelchair robots creates opportunities for elderly people to interact with others by increasing opportunities to go outside. Such interactions are critical to enhance the motivation for rehabilitation in elderly by increase their social activities. The balance must be considered between using such moving support systems and rehabilitation, but a moving support system will help both caregivers and seniors.

### Novelty and habituation effects

In our experiment, since the elderly participants only used the wheelchair robot a few times, a novelty effect probably exists; if they get accustomed to it, the effects will change. For example, during the experiments, most of the seniors seemed interested in the call behaviors, but they will probably become inured to them. If the robot used place-oriented behaviors every time, some might feel annoyed. Perceived enjoyment might decrease through long-term interaction with the robot.

To moderate habituation effects, the robot must change its language during long-term use to avoid negative impressions by repeating the same scripts. Since people are likely to be accustomed to the moving speed of the wheelchair robot, it must learn changes in preferred speed through long-term use. In this experiment we could not reach out to such long-term problems because it is out of focus of the paper, but considering habituation effects would be important future works of the paper.

### Limitation

Since this study was only conducted for moving support at a single experimental residential care home in Japan, we cannot generalize about it.

In this experiment, we extracted single item from measuremtns (e.g., intention to use) to investigate the feeling of participants. Even though the validity of original scales was evaluated in the referred work [8] and the selection of only one item from scales was needed to conduct experiment with elderly participants who need daily care, this would be one weak point of our paper. A use of all items of the scales, or other common measurements such as Usefulness, Satisfaction, and Ease of use(USE) or Component-Based Usability Questionnaire(CBUQ) would be one of important future works of the paper.

We did not evaluate each social behavior by separating the conditions because the participants and the staff members from the care homes requested that we shorten the experiment as much as possible due to the physical condition of the participants. We also thought that the robot would use all of its social behaviors during the interactions because the human caregivers were already doing this in real situations. But, though this limitation complicates separation of the effects of each social behavior, we believe that the experimental results still provide enough knowledge, even if the effects of the implemented social behaviors are evaluated at once.

We also did not investigate the effects of different control methods of wheelchair robot and assumptions of elderly people towards the robot. In particular for a passing by situation with others, naturalness of avoiding behaviors is one of the important point for mobile robots [32]. Even if the elderly people did not know the wheelchair robot is controlled by itself or human operator, they might feel different impressions [33]. These research questions are different from current research questions of the paper, but investigation of them would be important to a use of wheelchair robots in real environments.

Another limitation is the variation of the environment. In this study we prepared a typical scene where elderly people use wheelchair in their daily life at resident homes, i.e., a round trip between their bed room and bathrooms. Even though such scenes include a situation where human caregivers socially interacted with elderly people who are using wheelchairs, considering of spatial variation, e.g., different room design or places like lobby area, common area or public spaces, might also be a relevant factor about social acceptance.

### Future work

This study showed strong evidence for continuing in the research direction of using robots for elderly support scenarios. This research direction will also help caregivers who support elderly people. One important future work is investigating whether the caregivers themselves prefer such robotics support for elderly people. Even if seniors want to use such a robotic support system, these systems cannot be installed in real environments without agreement from caregivers. Therefore, we will next conduct a similar study to investigate whether caregivers prefer such wheelchair robots that engage in social behaviors. Because, in this study we only learned that social behaviors from caregivers are accepted by elderly people for a wheelchair robot; but we did not design/investigate the effects of social behaviors which increase social acceptance for caregivers.

## Conclusion

This paper investigated whether elderly people prefer a wheelchair robot than human caregivers for moving support, by complementing locomotion with social behaviors which are designed based on observations of human caregivers in real environments. We developed an autonomous wheelchair robot and implemented the social behaviors, and then conducted experiments to investigate social acceptance of the robot from viewpoint of elderly people. Experimental results showed that elderly people highly evaluated the wheelchair robot with social behaviors more than human caregivers and the wheelchair robot without social behaviors. Interview results showed why elderly people preferred the wheelchair robot, e.g., the robot was easy to make a request for elderly people.

## Appendix

The preliminary stages of this research have been presented at the Human Agent Interaction (HAI 2014) conference, and published as part of the conference proceedings [30]. Therefore the part of data was collected in the past article, but we gathered extra data and analyzed them such as interview results. Moreover, the past paper aimed to develop the autonomous wheelchair system, different from the main contributions of this paper. As a result, the present work largely extends and completes the research that had been performed for the conference paper. The improvements include an investigation of perception of participants towards our robots, new hypothesis, prediction and discussion with deeper analysis.

## Supporting Information

S1 Table Questionnaire results. (XLSX)

S2 Table Coding results of interview data. (XLSX)
